# Association Between Human Leukocyte Antigen Class I and II Diversity and Non-virus-associated Solid Tumors

**DOI:** 10.3389/fgene.2021.675860

**Published:** 2021-08-04

**Authors:** Zhiwei Liu, Allan Hildesheim

**Affiliations:** Division of Cancer Epidemiology and Genetics, National Cancer Institute, Bethesda, MD, United States

**Keywords:** association, Caucasian, human leukocyte antigen, solid tumor, zygosity

## Abstract

Homozygosity at human leukocyte antigen (HLA) loci might lead to reduced immunosurveillance and increased disease risk, including cancers caused by infection or of hematopoietic origin. To investigate the association between HLA zygosity and risk of non-virus-associated solid tumors, we leveraged genome-wide association study (GWAS) data from over 28,000 individuals of European ancestry who participated in studies of 12 cancer sites (bladder, brain, breast, colon, endometrial, kidney, lung, ovary, pancreas, prostate, skin, and testis). Information on HLA zygosity was obtained by imputation; individuals were classified as homozygotes at a given locus when imputed to carry the same four-digit allele at that locus. We observed no evidence for an association between zygosity at six HLA loci and all cancers combined. Increase in number of homozygous at HLA class I loci, class II loci, or class I and II loci was also not associated with cancer overall (*P*_*trend*_ = 0.28), with adjusted odds ratios (ORs) for risk-per-locus of 1.00 [95% confidence intervals (CIs) = 0.97, 1.03], 1.02 (0.99, 1.04), and 1.01 (0.99, 1.02), respectively. This study does not support a strong role for HLA zygosity on risk of non-virus-associated solid tumors.

## Introduction

Immunosurveillance plays an important role in the body’s natural defense against the development of cancer. Human leukocyte antigens (HLA) are essential in the immunosurveillance hypothesis given its role in adaptive and innate immunity. Individuals who are heterozygous at the HLAs are better able to present a greater variety of antigenic peptides than those who are homozygous, resulting in an immune response to a broader range of antigens (Penn et al., 2002; Martin and Carrington, 2013). Studies of infectious diseases and cancers caused by viral infections and/or that arise within immune cells have demonstrated an association between HLA homozygosity and these outcomes (Thursz et al., 1997; Carrington et al., 1999; Wang et al., 2018). Reduced diversity may result in reduced antigen presentation, thus facilitating immune evasion and increasing disease risk. For example, homozygous individuals infected with hepatitis B virus (HBV) or hepatitis C virus (HCV) are more likely to have persistent infection than heterozygous individuals (Thursz et al., 1997; Hraber et al., 2007). After infection with human immunodeficiency virus (HIV), more rapid progression to acquired immunodeficiency syndrome (AIDS) was observed among homozygous individuals than heterozygous individuals (Carrington et al., 1999). Additional support for this hypothesis comes from studies of cancers caused by infections (Liu et al., 2020) or of hematopoietic origin [e.g., certain subtypes of non-Hodgkin lymphoma (NHL)] (Wang et al., 2010, 2018).

However, to our knowledge, the association between HLA diversity and risk of solid tumors that have not been linked to a viral infection remains unclear. According to the immunosurveillance hypothesis, HLA variations may affect the ability of each individual’s immune system to recognize tumor-associated antigens and neoantigens that are expressed by non-viral infection-related solid organ tumors. Heterozygous HLA genotypes should facilitate presentation of these antigens resulting in stronger cytotoxic T lymphocyte response, thereby reducing risk of solid organ tumors. To test our hypothesis, here, we utilized genome-wide association study (GWAS) data from individuals of European ancestry who participated in large GWAS genotyped at or reported to the National Cancer Institute’s Cancer Genomics Research Laboratory between 2007 and 2014, to investigate the association between HLA zygosity at class I and class II loci and risk of solid tumors at 12 different sites.

## Materials and Methods

### Study Population

The present study is composed of 29,343 subjects of European ancestry who participated in 10 studies across 12 cancer sites as primary diagnoses, including cancers of the bladder, brain, breast, colon, endometrial, kidney, lung, ovary, pancreas, prostate, skin, and testis, which have been described in detail elsewhere (Liu et al., 2021; Supplementary Material and Supplementary Table 1). Cases selected were either histopathologically confirmed or identified through linkage to the Cancer Registers as primary cancers. Participants in these studies provided written informed consent and were enrolled in institutional review board (IRB)-approved projects.

### Genotyping and Quality Control

Genome-wide association study platforms used include the Illumina Human1M-Duo BeadChip, Illumina Human Omni2.5-8 BeadChip, Illumina HumanHap 550K, Illumina HumanHap 610K, Illumina HumanHap 660W, Illumina HumanOmni1, and Illumina OmniExpress. Quality control was performed within each study and has been described in detail elsewhere (Liu et al., 2021). Briefly, we excluded single-nucleotide polymorphisms (SNPs) with missing rate > 10% and subjects with missing SNPs > 10% (*N* = 366) and subjects with missing information on sex and/or age at diagnosis (*N* = 104). In addition, we excluded seven subjects diagnosed with skin cancer in the ATBC study and two subjects scanned using Illumina HumanOmni1. Finally, to evaluate population substructure, a principal component analysis (PCA) was performed using PLINK (version 1.9). Plots of the first two principal components for each study are shown in Supplementary Figure 1. We excluded 11 outliers, resulting in a total of 28,853 subjects (17,405 cases and 11,448 controls) in the final analysis.

### Human Leukocyte Antigen Imputation

After quality control steps, we used the genotyping data from 25,759,242 to 33,534,827 bp at chromosome 6 based on hg19 positions to impute classical HLA alleles at three HLA class I loci (HLA-A, B, and C) and four HLA class II loci (HLA-DQA1, DQB1, DRB1, and DPB1) at the four-digit allele level by “HIBAG” in R programming (Zheng et al., 2014). The reference panel used for imputation included 2,668 of individuals of European descent included in GlaxoSmithKline (GSK) clinical trials (Zheng et al., 2014). Validation of imputation accuracy was performed in an independent set of 640 samples by comparing imputed HLA alleles with four-digit HLA sequencing data for HLA-A, B, C, and DRB1 loci processed using the same protocol used in the main study. We found high concordance on four-digit allele calling at all loci (>94% for HLA-A, B, C, and DRB1) and even higher concordance on homozygosity calling for these same loci (>98%, Supplementary Table 2). Individuals were classified as homozygotes at a given locus when imputed to carry the same four-digit allele at that locus.

### Statistical Analysis

Odds ratios (ORs) and 95% confidence intervals (CIs) were estimated by logistic regression for the association between homozygosity at each individual HLA locus and the number of homozygous loci for class I and class II and risk of individual cancers. Separate logistic regression models were developed for each cancer site within each individual study. All models were adjusted for sex, age, and population structure (top 10 principal components). Tests for linear trend were conducted using ordinal variables for number of homozygous loci for class I, class II, and class I and II combined. Based on study-specific ORs and standard errors estimated from the individual’s logistic regression models described above, fixed-effects meta-analysis was used to combine individual within-study association estimates from cancers included in at least two studies and pan-cancer analysis. For each *P* trend, we also present the linearized additive risk-per-locus, reflecting the slope of the trend line. Genetic effect heterogeneity across studies was assessed using the I^2^ statistic and the *P* value for heterogeneity calculated from Cochran’s Q statistic. In sensitivity analyses, we restricted to a post-imputation quality control call threshold of >0.5, as recommended by the HIBAG package (Zheng et al., 2014).

Statistical analyses were performed using R statistical software^1^. The statistical tests were two tailed with α = 0.05. For trend tests, results were considered significant if the *P* was < 0.002 (Bonferroni correction for multiple testing with 12 cancer types and two HLA loci evaluated, 0.05/24).

### Power Calculation

For the primary aim of assessing the association between HLA zygosity at two HLA loci and non-virus-associated solid tumors overall, with ∼17,000 cases and ∼11,000 controls from 10 studies, we had at least 80% power to detect an OR as low as 1.07, assuming α = 0.025 (Bonferroni correction for multiple testing with two HLA loci evaluated, 0.05/2).

## Results

Supplementary Table 1 and Table 1 shows the numbers of cases and controls of European ancestry from each of the 10 studies in North America and Europe. The number of cases ranged from 68 (for skin cancer) to 4,984 (for prostate cancer). Cases and controls had similar age and sex distributions (Supplementary Table 3). The prevalence of homozygosity was also similar for cases and controls (Supplementary Tables 4–15).

**TABLE 1:** Associati between homozygosity at HLA class I loci and class II loci and risk of cancer.^a^

Cancer	No of studies	Cases	Controls	Class I locus			Class II locus			Class I + Class II loci		
		
		*n*	*n*	OR_2+*vs.none*_^b^	OR per locus^c^	*P*_*trend*_^d^	OR_2+*vs.none*_^b^	OR per locus^c^	*P*_*trend*_^d^	OR_2+*vs.none*_^b^	OR_per locus_^c^	*P*_*trend*_^d^
Total	10	17,405	11,448 ^e^	1.06 (0.97, 1.16)	1.00 (0.97, 1.03)	0.98	1.04 (0.98, 1.11)	1.02 (0.99, 1.04)	0.16	1.00 (0.95, 1.06)	1.01 (0.99, 1.02)	0.28
Bladder	4	3,020	7,422	0.95 (0.77, 1.16)	0.95 (0.88, 1.03)	0.19	0.89 (0.77, 1.04)	0.97 (0.92, 1.02)	0.18	0.90 (0.80, 1.02)	0.97 (0.93, 1.00)	0.07
Brain	3	446	5,648	1.01 (0.61, 1.67)	0.95 (0.79, 1.13)	0.54	1.13 (0.81, 1.58)	1.05 (0.94, 1.17)	0.41	1.06 (0.79, 1.42)	1.02 (0.93, 1.11)	0.70
Breast	2	968	4,288	1.27 (0.86, 1.86)	1.06 (0.92. 1.23)	0.39	1.08 (0.82, 1.43)	1.02 (0.93, 1.12)	0.66	0.99 (0.78, 1.26)	1.03 (0.96, 1.11)	0.37
Colon	2	82	5,263	1.42 (0.64, 3.18)	1.11 (0.81, 1.52)	0.52	0.96 (0.45, 2.05)	1.00 (0.80, 1.25)	0.99	1.12 (0.63, 2.00)	1.03 (0.87, 1.21)	0.76
Endometrial	2	855	3,877	1.19 (0.69, 2.03)	1.05 (0.87, 1.26)	0.64	1.17 (0.80, 1.70)	1.02 (0.90, 1.15)	0.74	1.13 (0.83, 1.53)	1.02 (0.93, 1.12)	0.63
Kidney	3	1,133	5,823	1.11 (0.84, 1.48)	1.03 (0.93, 1.15)	0.58	1.10 (0.87, 1.38)	1.02 (0.95, 1.10)	0.56	1.08 (0.89, 1.30)	1.02 (0.96, 1.08)	0.51
Lung	3	4,748	7,253	1.04 (0.88, 1.23)	0.99 (0.93, 1.06)	0.83	1.05 (0.94, 1.19)	1.03 (0.99, 1.07)	0.18	0.99 (0.89, 1.09)	1.01 (0.98, 1.04)	0.36
Ovary	1	264	3,814	0.98 (0.50, 1.92)	0.96 (0.76, 1.21)	0.73	1.32 (0.84, 2.08)	1.10 (0.95, 1.27)	0.20	1.11 (0.75, 1.63)	1.05 (0.94, 1.17)	0.39
Pancreas	2	391	5,263	1.24 (0.84, 1.85)	1.02 (0.87, 1.20)	0.77	1.05 (0.75, 1.48)	1.00 (0.90, 1.12)	0.94	1.07 (0.81, 1.41)	1.01 (0.93, 1.09)	0.80
Prostate	2	4,982	5,263	1.07 (0.90, 1.27)	1.03 (0.96, 1.09)	0.44	1.10 (0.96, 1.26)	1.04 (0.99, 1.08)	0.11	1.07 (0.96, 1.20)	1.03 (0.99, 1.06)	0.11
Skin	1	68	3,814	0.96 (0.34, 2.69)	0.87 (0.57, 1.33)	0.52	1.37 (0.68, 2.75)	1.05 (0.82, 1.34)	0.69	0.91 (0.48, 1.72)	1.00 (0.82, 1.22)	1.00
Testis	1	448	554	1.08 (0.62, 1.88)	0.97 (0.79, 1.20)	0.78	0.88 (0.60, 1.30)	0.93 (0.82, 1.06)	0.26	0.80 (0.57, 1.12)	0.95 (0.86, 1.05)	0.32

*CI, confidence interval; HLA, human leukocyte antigen. ^a^Odds ratios (ORs) were obtained from the meta-analysis summarizing estimates based on unconditional logistic regression models adjusted for sex (if possible), age, and top 10 PCs. For cancer with only one study included, ORs were obtained from unconditional logistic regression models. ^b^ORs for homozygous for two or more loci vs. zygosity for all loci. ORs were obtained from the meta-analysis. ^c^ORs for one increase number of homozygous loci. ORs were obtained from the meta-analysis. ^d^Two-sided P values for trend were assessed using ordinal number of the homozygous loci with 1 degree of freedom, which were obtained from the meta-analysis if more than one study were included. ^e^Number of unique controls included in the study.*

### Association With Increasing Number of Homozygotes

The association between number of homozygous loci at HLA class I, class II, and class I/class II combined for cancer overall and individual cancer sites is summarized in Table 1 and Supplementary Tables 4–15. Increase in number of homozygous HLA class I loci, class II loci, or class I and II loci together was not associated with cancer overall, with adjusted ORs (95% CI) for risk-per-locus of 1.00 (0.97, 1.03), 1.02 (0.99, 1.04), and 1.01 (0.99, 1.02), respectively (Table 1). Individuals with two or more homozygous class I loci, class II loci, or class I and II loci together were not associated with cancer overall, compared with heterozygosity at these loci. When the association between increase in number of homozygous HLA loci and individual cancer sites was evaluated, no significant associations were observed (Table 1).

### Association With Zygosity at Individual Human Leukocyte Antigen Loci

The associations between zygosity at HLA class I loci (A, B, and C) and class II loci (DRB1, DPB1, and DQA1) and cancer (overall and for individual cancer sites) are summarized in Table 2 and Supplementary Tables 4–15. A nominally significant association with cancer overall was observed for homozygosity at HLA-DQB1 locus (adjusted OR = 1.08, 95% CI = 1.01–1.15, *P* = 0.016, Table 2). However, there were no significant associations between zygosity at HLA class I loci (A, B, and C) and other class II loci (DRB1, DPB1, and DQA1) and cancer overall. For individual cancer sites, we only observed nominally significant associations with breast cancer for homozygosity at HLA-C locus (adjusted OR = 1.38, 95% CI = 1.02–1.87, *P* = 0.036, Supplementary Table 6) and lung cancer for homozygosity at HLA-DQB1 locus (adjusted OR = 1.15, 95% CI = 1.02–1.29, *P* = 0.022, Supplementary Table 10). For cancer sites included in two or more studies, no significant heterogeneity was observed across studies (all *P* values for heterogeneity > 0.002).

**TABLE 2:** Association between homozygosity at HLA class I loci and class II loci and risk of all cancers in Caucasian participants within NCI genome-wide association studies.

		Cases (*n* = 17,405) *n* (%)	Controls (*n* = 11,448) *n* (%)	OR (meta-analysis, 95% CI)	*P*-heterogeneity
**Class I locus**					
HLA-A	Homozygote	2,576(14.8)	1,694(14.8)	0.97(0.91,1.02)	0.79
HLA-B	Homozygote	1,190(6.8)	762 (6.7)	1.02(0.95,1.11)	0.73
HLA-C	Homozygote	1,673(9.6)	1,077(9.4)	1.04(0.97,1.12)	0.63
**Class II locus**					
HLA-DRB1	Homozygote	1,575(9.0)	1,012(8.8)	1.04(0.97,1.12)	0.68
HLA-DPB1	Homozygote	4,291(24.7)	2,760(24.1)	1.00(0.96,1.05)	0.97
HLA-DQA1	Homozygote	2,269(13.0)	1,502(13.1)	1.02(0.96,1.09)	0.76
HLA-DQB1	Homozygote	2,098(12.1)	1,355(11.8)	1.08(1.01,1.15)	0.23

*CI, confidence interval; HLA, human leukocyte antigen; NCI, National Cancer Institute; OR, odds ratio.*

In sensitivity analysis, we did not observe an association between zygosity at HLA loci and any of the 12 cancers evaluated. For example, increase in number of homozygous HLA class I loci, class II loci, or class I and II loci altogether was not associated with cancer overall, with the adjusted ORs (95% CI) for risk-per-locus of 0.99 (0.96, 1.03), 1.02 (1.00, 1.04), and 1.01 (0.99, 1.02), respectively.

## Discussion

It is hypothesized that HLA homozygosity leads to reduced immunosurveillance and consequently to an increased risk of diseases including cancers. However, in the present study that evaluated cancers that are not caused by viral infection and that do not arise in immune cells, we observed little evidence for an increase in risk of non-virus-associated solid tumors or 12 types individually with HLA homozygosity. Of note, even for cancers that typically have a high mutational load (i.e., higher number of neoepitopes) or associated with individuals SNPs within the HLA region, such as lung cancer (Efremova et al., 2017; Liu et al., 2021), no association with HLA homozygosity was noted.

Diversity of antigen presentation by the HLA is constrained in a haplotype- and allele-specific manner. Statistically, a higher diversity among HLA loci is related to a higher diversity of antigen presentation. It also could be true for non-infectious neoplasms given that the immune system is capable of recognizing and even eradicating cancerous cells based on the antigenic repertoire that differentiates neoplastic self from non-neoplastic self (e.g., neoantigen). However, our data do not support this hypothesis. We observed no association between HLA homozygosity and non-infection-related solid organ tumors, regardless of cancer types with different tumor mutational loads, which are correlated with number of neoepitopes (Efremova et al., 2017). The underlying mechanisms are unclear. It is plausible to hypothesize that HLA is more efficient in binding exogenous antigens rather than neoantigens during tumor development (Chowell et al., 2019). Alternatively, viruses are known to active high-affinity antigen-specific cytotoxic T cells, while the immune cytolytic activities induced by neoantigens are weaker (Rooney et al., 2015). Therefore, although the mutations yielding predicted by HLA-binding peptides could be higher among HLA heterozygotes, the level of cytolytic activity mounted by antigen presentation is not enough to control tumor development (Rooney et al., 2015). Our data suggest that impact of reduced immunosurveillance due to HLA homozygosity seems to be restricted to cancers that present exogenous antigens or where the immune cells themselves are the target of the cancer.

In addition to the null association between HLA zygosity and non-virus-associated solid tumor overall, we observed a nominally significant association between homozygosity at HLA-C locus and breast cancer and homozygosity at HLA-DQB1 locus and lung cancer. False-positive findings cannot be ruled out given that the association is not significant after correction for multiple testing. Further investigation is warranted to confirm or refute the observed associations.

Our study strengths include the large sample size overall from multiple studies. We observed no evidence of heterogeneity of associations across studies, which further allay concerns of false-negative findings. However, our study had several limitations. Our models did not include variables associated with environmental or lifestyle or disease-related factors. However, it is unlikely that the association between HLA zygosity, which is considered as a germline genetic variant, and cancer can be confounded by those factors. Due to modest sample size for some individual cancer types, we may have had limited power to identify modest cancer-specific associations (e.g., for cancers of colon and skin). For several common cancers (e.g., cancers of lung, breast, colon, and skin), future studies with larger sample sizes and more advanced approach for controlling false discovery rate (Ge et al., 2020) are warranted to detect a modest effect of HLA zygosity.

In conclusion, our data suggest that impact of reduced immunosurveillance due to HLA homozygosity seems to be restricted to cancers that present exogenous antigens or where the immune cells themselves are the target of the cancer. Nevertheless, zygosity is just one ramification of the diversity of HLA allele at class I or II loci; structural variants, HLA expression, and other characteristics of these loci are also necessary to probe in order to fully understand the impact of HLA variation on cancer immunosurveillance. Increasing our sample size in future studies might provide greater power to detect a modest association between HLA zygosity and a specific type of non-virus-associated solid tumors.

## Data Availability Statement

The original contributions presented in the study are included in the article/Supplementary Material, further inquiries can be directed to the corresponding author/s.

## Ethics Statement

The studies involving human participants were reviewed and approved by the National Cancer Institute. The patients/participants provided their written informed consent to participate in this study.

## Author Contributions

ZL designed and oversaw the study and contributed to data analysis and manuscript writing. AH designed and oversaw the study and contributed to result interpretation and manuscript writing. Both authors contributed to the article and approved the submitted version.

## Conflict of Interest

The authors declare that the research was conducted in the absence of any commercial or financial relationships that could be construed as a potential conflict of interest.

## Publisher’s Note

All claims expressed in this article are solely those of the authors and do not necessarily represent those of their affiliated organizations, or those of the publisher, the editors and the reviewers. Any product that may be evaluated in this article, or claim that may be made by its manufacturer, is not guaranteed or endorsed by the publisher.

## Footnotes

1
http://www.r-project.org/
