# Supplementary material for: Association Between Human Leukocyte Antigen Class I and II Diversity and Non-virus-associated Solid Tumors
Source: Front Genet. 2021 Aug 4;12:675860. doi: 10.3389/fgene.2021.675860 (PMC8371526; doi:10.3389/fgene.2021.675860)
Supplement: Supplementary file 2 [file Presentation_1.pdf]

Supplementary Materials

Study Populations

The study includes individuals who participated in large case/control GWAS that were genotyped at or reported to the National Cancer Institute's Cancer Genomics Research Laboratory. GWAS data from 10 studies were obtained from different Illumina SNP genotyping arrays, including cases/controls of bladder cancer from 4 studies totaling 3,100 cases and 8,278 controls (1), brain cancer from 3 studies totaling 478 cases and 6516 controls (2), breast cancer from 2 studies totaling 1071 cases and 1325 controls (3), colon cancer from 2 studies totaling 122 cases and 6094 controls (4), endometrial cancer from 2 studies totaling 940 cases and 887 controls (5), kidney cancer from 3 studies totaling 1566 cases and 7056 controls (6), lung cancer from 3 studies totaling 4938 cases and 8086 controls (7), ovarian cancer from 1 study totaling 535 cases and 815 controls (8), pancreatic cancer from 2 studies totaling 425 cases and 6094 controls (9), prostate cancer from 2 studies totaling 5872 cases and 5279 controls (10), skin cancer from 2 studies totaling 99 cases and 6904 controls, and testicular cancer from one study totaling 531 cases and 637 controls (11). Some individuals were included as controls in multiple GWAS and, in total, there were 12,873 unique individuals included as controls. We only included individuals with European ancestry, totaling 17,693 cases and 11,650 unique controls in the analysis.

Reference

1. Figueroa JD, Ye Y, Siddiq A, Garcia-Closas M, Chatterjee N, Prokunina-Olsson L*, et al.* Genome-wide association study identifies multiple loci associated with bladder cancer risk. Hum Mol Genet **2014**;23:1387-98
2. Rajaraman P, Melin BS, Wang Z, McKean-Cowdin R, Michaud DS, Wang SS*, et al.* Genome-wide association study of glioma and meta-analysis. Hum Genet **2012**;131:1877-88

3. Siddiq A, Couch FJ, Chen GK, Lindstrom S, Eccles D, Millikan RC*, et al.* A meta-analysis of genome-wide association studies of breast cancer identifies two novel susceptibility loci at 6q14 and 20q11. Hum Mol Genet **2012**;21:5373-84
4. Major JM, Yu K, Wheeler W, Zhang H, Cornelis MC, Wright ME*, et al.* Genome-wide association study identifies common variants associated with circulating vitamin E levels. Hum Mol Genet **2011**;20:3876-83
5. De Vivo I, Prescott J, Setiawan VW, Olson SH, Wentzensen N, Attia J*, et al.* Genome-wide association study of endometrial cancer in E2C2. Hum Genet **2014**;133:211-24
6. Purdue MP, Johansson M, Zelenika D, Toro JR, Scelo G, Moore LE*, et al.* Genome-wide association study of renal cell carcinoma identifies two susceptibility loci on 2p21 and 11q13.3. Nat Genet **2011**;43:60-5
7. Landi MT, Chatterjee N, Yu K, Goldin LR, Goldstein AM, Rotunno M*, et al.* A genome-wide association study of lung cancer identifies a region of chromosome 5p15 associated with risk for adenocarcinoma. American journal of human genetics **2009**;85:679-91
8. Wentzensen N, Black A, Jacobs K, Yang HP, Berg CD, Caporaso N*, et al.* Genetic variation on 9p22 is associated with abnormal ovarian ultrasound results in the Prostate, Lung, Colorectal, and Ovarian Cancer Screening Trial. PLoS One **2011**;6:e21731
9. Wolpin BM, Rizzato C, Kraft P, Kooperberg C, Petersen GM, Wang Z*, et al.* Genome-wide association study identifies multiple susceptibility loci for pancreatic cancer. Nat Genet **2014**;46:994-1000
10. Schumacher FR, Berndt SI, Siddiq A, Jacobs KB, Wang Z, Lindstrom S*, et al.* Genome-wide association study identifies new prostate cancer susceptibility loci. Hum Mol Genet **2011**;20:3867-75
11. Chung CC, Kanetsky PA, Wang Z, Hildebrandt MA, Koster R, Skotheim RI*, et al.* Meta-analysis identifies four new loci associated with testicular germ cell tumor. Nat Genet **2013**;45:680-5

Supplementary Table 1. Genome wide association studies (GWAS) included in the evaluation of HLA homozygosity and risk of cancer

Study name	Study abbreviation	Cases (n=17,405)												Controls
		Bladder (n=3020)	Brain (n=446)	Breast (n=968)	Colon (n=82)	Endometrial (n=855)	Kidney (n=1133)	Lung (n=4748)	Ovary (n=264)	Pancreas (n=391)	Prostate (n=4982)	Skin (n=68)	Testis (n=448)	(n=11,448)
Alpha-Tocopherol, Beta-Carotene Lung Cancer Prevention Study	ATBC	366	32	0	22	0	170	1590	0	174	366	0	0	1449
The Environment And Genetics in Lung cancer Etiology	EAGLE	0	0	0	0	0	0	1926	0	0	0	0	0	1990
NCI Brain Tumor Study	NCI_BTS	0	305	0	0	0	0	0	0	0	0	0	0	385
New England Bladder Cancer	NEBL	969	0	0	0	0	0	0	0	0	0	0	0	1114
Polish Breast Cancer Study	PBCS	0	0	641	0	0	0	0	0	0	0	0	0	474
Polish Endometrial Cancer Study	PECC	0	0	0	0	408	0	0	0	0	0	0	0	63
Prostate, Lung, Colorectal, and Ovarian Cancer Screening Trial	PLCO	579	109	327	60	447	316	1232	264	217	4616	68	0	3814
Spanish Bladder Cancer study	SPBC	1106	0	0	0	0	0	0	0	0	0	0	0	1045
the US Servicemen's Testicular Tumor Environmental and Endocrine Determinants	STEED	0	0	0	0	0	0	0	0	0	0	0	448	554
US Renal Cancer Study	USRC	0	0	0	0	0	647	0	0	0	0	0	0	560

Supplementary Table 2. Concordances for comparison of imputed human leukocyte antigen (HLA) alleles to 4-digit HLA sequencing data available for a subset of samples.

	HLA-A (n=635)	HLA-B (n=637)	HLA-C (n=640)	HLA-DRB1 (n=605)
Allele	97.0%	96.1%	98.3%	94.2%
Homozygosity	99.5%	99.4%	98.0%	98.5%

Supplementary Table 3. Distributions on sex and age at diagnosis/sample collection by case-control status.

	**Cases (n=17405) n (%)**	**Controls (n=11448) n (%)**
**Sex**		
Female	4139 (23.8)	2357 (20.6)
Male	13266 (76.2)	9091 (79.4)
**Age at diagnosis/sample collection (years)**		
<30	401 (2.3)	497 (4.3)
30-39	230 (1.3)	298 (2.6)
40-49	559 (3.2)	533 (4.7)
50-59	2508 (14.4)	1862 (16.3)
60-69	8234 (47.3)	5095 (44.5)
70-79	5315 (30.5)	3099 (27.1)
80+	158 (0.9)	64 (0.6)

Supplementary Table 4. Association between homozygosity at HLA class I gene and class II gene and risk of bladder cancer.

		**Bladder (n=3020) n (%)**	**Controls (n=7422) n (%)**	**OR (95% CI)**	**p-heterogeneity**
**Class I locus**					
HLA-A	Homozygote	383 (12.7)	1155 (15.6)	0.86 (0.75, 0.98)	0.30
HLA-B	Homozygote	193 (6.4)	514 (6.9)	1.00 (0.83, 1.21)	0.29
HLA-C	Homozygote	267 (8.8)	680 (9.2)	0.99 (0.85, 1.17)	0.48
**Class II locus**					
HLA-DRB1	Homozygote	238 (7.9)	644 (8.7)	0.89 (0.75, 1.05)	0.77
HLA-DPB1	Homozygote	710 (23.5)	1811 (24.4)	0.99 (0.89, 1.10)	0.85
HLA-DQA1	Homozygote	353 (11.7)	909 (12.2)	0.90 (0.78, 1.04)	0.72
HLA-DQB1	Homozygote	321 (10.6)	827 (11.1)	0.91 (0.79, 1.05)	0.27

CI=confidence interval; HLA=human leukocyte antigen; NCI=National Cancer Institute; OR=odds ratio

Supplementary Table 5. Association between homozygosity at HLA class I loci and class II loci and risk of brain cancer in Caucasian participants within NCI genome-wide association studies.

		Brain (n=446) n (%)	Controls (n=5648) n (%)	OR (95% CI)	p-heterogeneity
**Class I locus**					
HLA-A	Homozygote	67 (15.0)	912 (16.1)	1.07 (0.78, 1.47)	0.70
HLA-B	Homozygote	23 (5.2)	413 (7.3)	0.76 (0.47, 1.24)	0.04
HLA-C	Homozygote	38 (8.5)	537 (9.5)	0.86 (0.58, 1.27)	0.01
**Class II locus**					
HLA-DRB1	Homozygote	38 (8.5)	492 (8.7)	0.96 (0.64, 1.42)	0.03
HLA-DPB1	Homozygote	121 (27.1)	1414 (25.0)	1.06 (0.82, 1.37)	0.64
HLA-DQA1	Homozygote	68 (15.2)	689 (12.2)	1.23 (0.90, 1.69)	0.06
HLA-DQB1	Homozygote	53 (11.9)	609 (10.8)	1.45 (0.81, 1.62)	0.01

CI=confidence interval; HLA=human leukocyte antigen; NCI=National Cancer Institute; OR=odds ratio

Supplementary Table 6. Association between homozygosity at HLA class I loci and class II loci and risk of breast cancer in Caucasian participants within NCI genome-wide association studies.

		Breast (n=968) n (%)	Controls (n=4288) n (%)	OR (95% CI)	p-heterogeneity
**Class I locus**					
HLA-A	Homozygote	125 (12.9)	616 (14.4)	0.93 (0.71, 1.22)	0.84
HLA-B	Homozygote	62 (6.4)	293 (6.8)	1.07 (0.74, 1.54)	0.19
HLA-C	Homozygote	107 (11.1)	386 (9.0)	1.38 (1.02, 1.87)	0.20
**Class II locus**					
HLA-DRB1	Homozygote	81 (8.4)	351 (8.2)	1.11 (0.80, 1.54)	0.79
HLA-DPB1	Homozygote	236 (24.4)	1063 (24.8)	0.94 (0.76, 1.16)	0.46
HLA-DQA1	Homozygote	128 (13.2)	526 (12.3)	1.14 (0.87, 1.49)	0.33
HLA-DQB1	Homozygote	112 (11.6)	462 (10.8)	1.13 (0.85, 1.50)	0.05

CI=confidence interval; HLA=human leukocyte antigen; NCI=National Cancer Institute; OR=odds ratio

Supplementary Table 7. Association between homozygosity at HLA class I loci and class II loci and risk of colon cancer in Caucasian participants within NCI genome-wide association studies.

		Colon (n=82) n (%)	Controls (n=5263) n (%)	OR (95% CI)	p-heterogeneity
**Class I locus**					
HLA-A	Homozygote	15 (18.3)	857 (16.3)	1.16 (0.66, 2.07)	0.47
HLA-B	Homozygote	7 (8.5)	381 (7.2)	1.23 (0.56, 2.72)	0.96
HLA-C	Homozygote	9 (11.0)	490 (9.3)	1.18 (0.58, 2.39)	0.63
**Class II locus**					
HLA-DRB1	Homozygote	7 (8.5)	450 (8.6)	0.96 (0.44, 2.12)	0.92
HLA-DPB1	Homozygote	18 (22.0)	1310 (24.9)	0.85 (0.50, 1.45)	0.92
HLA-DQA1	Homozygote	12 (14.6)	631 (12.0)	1.26 (0.67, 2.37)	0.53
HLA-DQB1	Homozygote	9 (11.0)	559 (10.6)	1.13 (0.56, 2.31)	0.17

CI=confidence interval; HLA=human leukocyte antigen; NCI=National Cancer Institute; OR=odds ratio

Supplementary Table 8. Association between homozygosity at HLA class I loci and class II loci and risk of endometrial cancer in Caucasian participants within NCI genome-wide association studies.

		Endometrial (n=855) n (%)	Controls (n=3877) n (%)	OR (95% CI)	p-heterogeneity
**Class I locus**					
HLA-A	Homozygote	128 (15.0)	561 (14.5)	1.17 (0.85, 1.62)	0.74
HLA-B	Homozygote	53 (6.2)	267 (6.9)	0.97 (0.60, 1.58)	0.34
HLA-C	Homozygote	74 (8.7)	352 (9.1)	0.96 (0.64, 1.43)	0.91
**Class II locus**					
HLA-DRB1	Homozygote	82 (9.6)	321 (8.3)	1.20 (0.80, 1.80)	0.61
HLA-DPB1	Homozygote	220 (25.7)	965 (24.9)	0.90 (0.69, 1.17)	0.88
HLA-DQA1	Homozygote	113 (13.2)	474 (12.2)	1.10 (0.77, 1.57)	0.44
HLA-DQB1	Homozygote	101 (11.8)	414 (10.7)	1.15 (0.79, 1.68)	0.48

CI=confidence interval; HLA=human leukocyte antigen; NCI=National Cancer Institute; OR=odds ratio

Supplementary Table 9. Association between homozygosity at HLA class I loci and class II loci and risk of kidney cancer in Caucasian participants within NCI genome-wide association studies.

		Kidney (n=1133) n (%)	Controls (n=5823) n (%)	OR (95% CI)	p-heterogeneity
**Class I locus**					
HLA-A	Homozygote	176 (15.5)	946 (16.2)	1.00 (0.83, 1.22)	0.38
HLA-B	Homozygote	83 (7.3)	422 (7.2)	1.04 (0.79, 1.36)	0.54
HLA-C	Homozygote	121 (10.7)	546 (9.4)	1.13 (0.89, 1.43)	0.92
**Class II locus**					
HLA-DRB1	Homozygote	104 (9.2)	502 (8.6)	1.03 (0.80, 1.33)	0.23
HLA-DPB1	Homozygote	287 (25.3)	1449 (24.9)	1.04 (0.88, 1.22)	0.65
HLA-DQA1	Homozygote	150 (13.2)	707 (12.1)	1.02 (0.82, 1.26)	0.55
HLA-DQB1	Homozygote	139 (12.3)	625 (10.7)	1.09 (0.87, 1.36)	0.25

CI=confidence interval; HLA=human leukocyte antigen; NCI=National Cancer Institute; OR=odds ratio

Supplementary Table 10. Association between homozygosity at HLA class I loci and class II loci and risk of lung cancer in Caucasian participants within NCI genome-wide association studies.

		Lung (n=4748) n (%)	Controls (n=7253) n (%)	OR (95% CI)	p-heterogeneity
**Class I locus**					
HLA-A	Homozygote	743 (15.6)	1103 (15.2)	0.97 (0.87, 1.08)	0.23
HLA-B	Homozygote	330 (7.0)	494 (6.8)	1.02 (0.88, 1.19)	0.85
HLA-C	Homozygote	467 (9.8)	692 (9.5)	1.00 (0.88, 1.14)	0.57
**Class II locus**					
HLA-DRB1	Homozygote	455 (9.6)	629 (8.7)	1.08 (0.95, 1.24)	0.43
HLA-DPB1	Homozygote	1132 (23.8)	1730 (23.9)	1.02 (0.93, 1.12)	0.74
HLA-DQA1	Homozygote	657 (13.8)	950 (13.1)	1.02 (0.91, 1.14)	0.29
HLA-DQB1	Homozygote	646 (13.6)	847 (11.7)	1.15 (1.02, 1.29)	0.93

CI=confidence interval; HLA=human leukocyte antigen; NCI=National Cancer Institute; OR=odds ratio

Supplementary Table 11. Association between homozygosity at HLA class I loci and class II loci and risk of ovarian cancer in Caucasian participants within NCI genome-wide association studies. [a]

		Ovary (n=264) n (%)	Controls (n=3814) n (%)	OR (95% CI)
**Class I locus**				
HLA-A	Homozygote	36 (13.6)	553 (14.5)	0.98 (0.64, 1.51)
HLA-B	Homozygote	15 (5.7)	265 (6.9)	0.81 (0.43, 1.51)
HLA-C	Homozygote	25 (9.5)	347 (9.1)	0.98 (0.59, 1.63)
**Class II locus**				
HLA-DRB1	Homozygote	32 (12.1)	317 (8.3)	1.37 (0.85, 2.21)
HLA-DPB1	Homozygote	32 (12.1)	317 (8.3)	1.10 (0.79, 1.54)
HLA-DQA1	Homozygote	32 (12.1)	317 (8.3)	1.23 (0.80, 1.89)
HLA-DQB1	Homozygote	32 (12.1)	317 (8.3)	1.28 (0.81, 2.02)

a Only one study was included in the analysis for Ovarian cancer; no p for heterogeneity was calculated.
CI=confidence interval; HLA=human leukocyte antigen; NCI=National Cancer Institute; OR=odds ratio

Supplementary Table 12. Association between homozygosity at HLA class I loci and class II loci and risk of pancreatic cancer in Caucasian participants within NCI genome-wide association studies.

		Pancreas (n=391) n (%)	Controls (n=5263) n (%)	OR (95% CI)	p-heterogeneity
**Class I locus**					
HLA-A	Homozygote	64 (16.4)	857 (16.3)	0.93 (0.70, 1.23)	0.46
HLA-B	Homozygote	33 (8.4)	381 (7.2)	1.17 (0.80, 1.72)	0.17
HLA-C	Homozygote	41 (10.5)	490 (9.3)	1.14 (0.81, 1.61)	0.37
**Class II locus**					
HLA-DRB1	Homozygote	36 (9.2)	450 (8.6)	1.05 (0.73, 1.51)	0.86
HLA-DPB1	Homozygote	95 (24.3)	1310 (24.9)	0.95 (0.74, 1.22)	0.13
HLA-DQA1	Homozygote	45 (11.5)	631 (12.0)	0.97 (0.70, 1.35)	0.79
HLA-DQB1	Homozygote	46 (11.8)	559 (10.6)	1.16 (0.84, 1.61)	0.68

CI=confidence interval; HLA=human leukocyte antigen; NCI=National Cancer Institute; OR=odds ratio

Supplementary Table 13. Association between homozygosity at HLA class I loci and class II loci and risk of prostate cancer in Caucasian participants within NCI genome-wide association studies.

		Prostate (n=4982) n (%)	Controls (n=5263) n (%)	OR (95% CI)	p-heterogeneity
**Class I locus**					
HLA-A	Homozygote	772 (15.5)	857 (16.3)	1.02 (0.90, 1.14)	0.20
HLA-B	Homozygote	359 (7.2)	381 (7.2)	1.05 (0.90, 1.24)	0.26
HLA-C	Homozygote	482 (9.7)	490 (9.3)	1.08 (0.93, 1.25)	0.96
**Class II locus**					
HLA-DRB1	Homozygote	455 (9.1)	450 (8.6)	1.10 (0.95, 1.27)	0.52
HLA-DPB1	Homozygote	1279 (25.7)	1310 (24.9)	1.05 (0.96, 1.16)	0.74
HLA-DQA1	Homozygote	640 (12.8)	631 (12.0)	1.07 (0.94, 1.21)	0.92
HLA-DQB1	Homozygote	571 (11.5)	559 (10.6)	1.08 (0.95, 1.24)	0.85

CI=confidence interval; HLA=human leukocyte antigen; NCI=National Cancer Institute; OR=odds ratio

Supplementary Table 14. Association between homozygosity at HLA class I loci and class II loci and risk of skin cancer in Caucasian participants within NCI genome-wide association studies. [a]

		Skin (n=68) n (%)	Controls (n=3814) n (%)	OR (95% CI)
**Class I locus**				
HLA-A	Homozygote	7 (10.3)	553 (14.5)	0.69 (0.31, 1.52)
HLA-B	Homozygote	5 (7.4)	265 (6.9)	1.11 (0.44, 2.80)
HLA-C	Homozygote	5 (7.4)	347 (9.1)	0.81 (0.32, 2.04)
**Class II locus**				
HLA-DRB1	Homozygote	7 (10.3)	317 (8.3)	1.29 (0.58, 2.85)
HLA-DPB1	Homozygote	16 (23.5)	948 (24.9)	0.92 (0.52, 1.62)
HLA-DQA1	Homozygote	8 (11.8)	467 (12.2)	0.98 (0.46, 2.07)
HLA-DQB1	Homozygote	10 (14.7)	408 (10.7)	1.44 (0.73, 2.85)

a Only one study was included in the analysis for Ovarian cancer; no p for heterogeneity was calculated.
CI=confidence interval; HLA=human leukocyte antigen; NCI=National Cancer Institute; OR=odds ratio

Supplementary Table 15. Association between homozygosity at HLA class I loci and class II loci and risk of testis cancer in Caucasian participants within NCI genome-wide association studies.

		Testis (n=448) n (%)	Controls (n=554) n (%)	OR (95% CI)
**Class I locus**				
HLA-A	Homozygote	60 (13.4)	78 (14.1)	0.94 (0.65, 1.35)
HLA-B	Homozygote	27 (6.0)	32 (5.8)	1.06 (0.62, 1.81)
HLA-C	Homozygote	37 (8.3)	48 (8.7)	0.92 (0.59, 1.45)
**Class II locus**				
HLA-DRB1	Homozygote	40 (8.9)	57 (10.3)	0.87 (0.57, 1.34)
HLA-DPB1	Homozygote	103 (23.0)	154 (27.8)	0.75 (0.56, 1.00)
HLA-DQA1	Homozygote	56 (12.5)	74 (13.4)	0.93 (0.64, 1.35)
HLA-DQB1	Homozygote	55 (12.3)	64 (11.6)	1.07 (0.73, 1.58)

a Only one study was included in the analysis for Ovarian cancer; no p for heterogeneity was calculated. CI=confidence interval; HLA=human leukocyte antigen; NCI=National Cancer Institute; OR=odds ratio

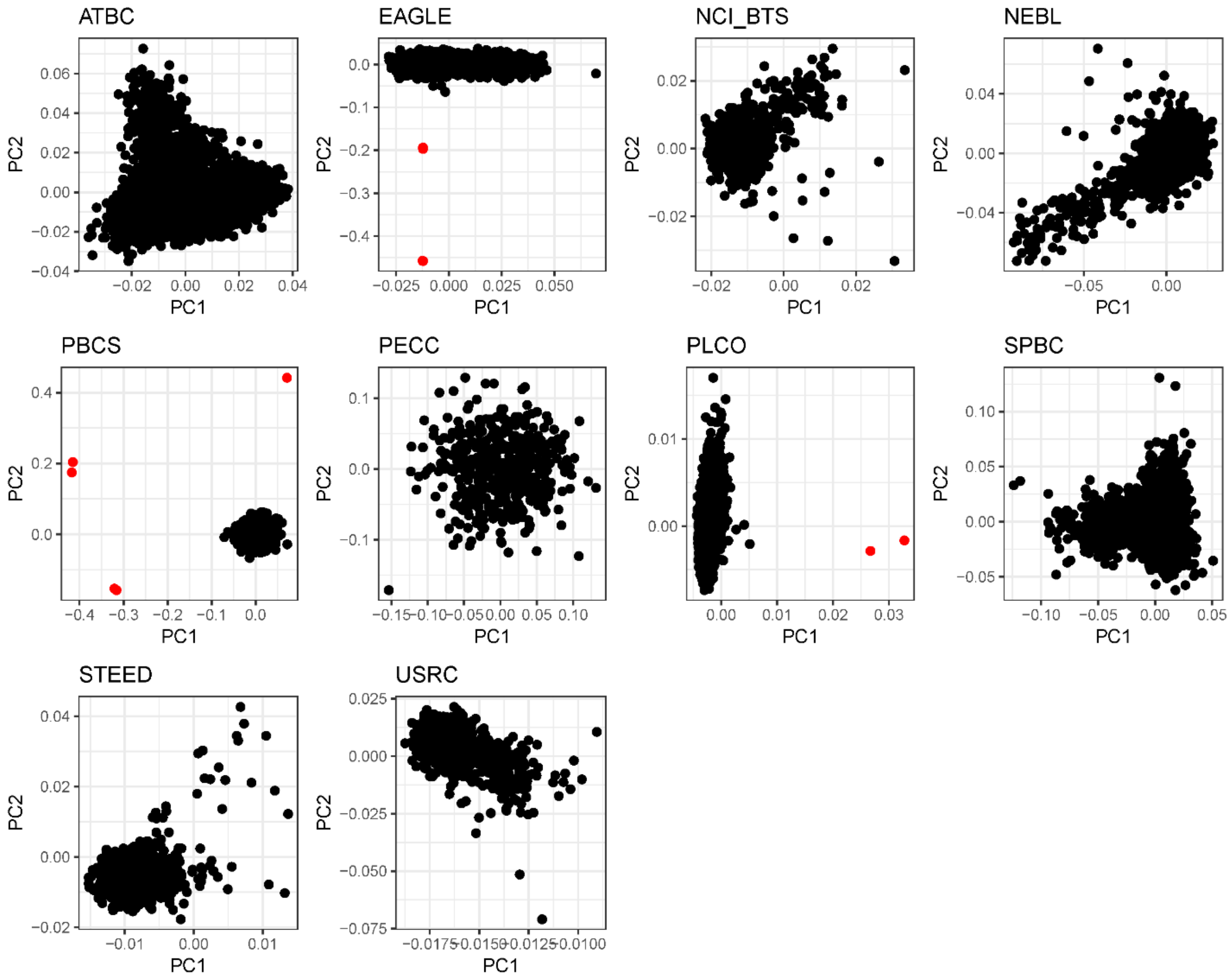

Supplementary Figure 1: Scatter plots based on first two principal components for ten GWAS studies included. Subjects excluded are indicated in red. ATBC=Alpha-Tocopherol, Beta-Carotene Lung Cancer Prevention Study; EAGLE=The Environment And Genetics in Lung cancer Etiology; NCI_BTS=NCI Brain Tumor Study; NEBL=New England Bladder Cancer; PBCS=Polish Breast Cancer Study; PECC=Polish Endometrial Cancer Study; PLCO=Prostate, Lung, Colorectal, and Ovarian Cancer Screening Trial; SPBC=Spanish Bladder Cancer study; STEED=the US Servicemen's Testicular Tumor Environmental and Endocrine Determinants; USRC=US Renal Cancer Study.
